# Triallyl Isocyanurate as an Efficient Electrolyte Additive for Layered Oxide Cathode Material-Based Lithium-Ion Batteries with Improved Stability under High-Voltage

**DOI:** 10.3390/molecules27103107

**Published:** 2022-05-12

**Authors:** Chang-Ming Zhang, Feng Li, Xue-Quan Zhu, Jin-Gang Yu

**Affiliations:** 1 College of Chemistry and Chemical Engineering, Central South University, Changsha 410083, China; maxwell.zhang@highpowertech.com; 2 Research Institute of Highpower International, Huizhou 516057, China; fli@highpowertech.com; 3 Shanshan Advanced Materials (QuZhou) Co., Ltd., Quzhou 324012, China; zhu.xuequan@shanshan.com

**Keywords:** multifunctional electrolyte additive, improved interface stability, triallyl isocyanurate

## Abstract

In this study, a new electrolyte additive 1,3,5-tri-2-propenyl-1,3,5-triazine-2,4,6-(1H, 3H, 5H)-trione (TAIC) for lithium-ion batteries is reported. The additive is introduced as a novel electrolyte additive to enhance electrochemical performances of layered lithium nickel cobalt manganese oxide (NCM) and lithium cobalt oxide (LiCoO_2_) cathodes, especially under a higher working voltage. Encouragingly, we found protective films would be formed on the cathode surface by the electrochemical oxidation, and the stability of the cathode material–electrolyte interface was greatly promoted. By adding 0.5 wt.% of TAIC into the electrolyte, the battery exhibited outstanding performances. The thickness swelling decreased to about 6% after storage at 85 °C for 24 h, while the capacity retention of cycle-life performances under high temperature of 45 °C after the 600th cycle increased 10% in comparison with the batteries without TAIC. Due to its specific function, the additive can be used in high energy density and high voltage lithium-ion battery systems.

## 1. Introduction

With the popularity of the 5G network, handheld terminals possessing more and more abundant functions are constantly emerging, and these multi-functional terminal devices require secondary batteries with higher capacity. Therefore, numerous flexible batteries with longer cycle life-time and lower life cycle cost are urgently needed. With the growing need for rechargeable batteries with relatively higher capacity and safety, numerous efforts have been made to develop new high-performance lithium-ion secondary batteries (LIBs) [1,2]. Layered lithium nickel cobalt manganese oxide (NCM) and lithium cobalt oxide (LiCoO_2_) materials have attracted much attention as a result of their high capacity, stable performance and high degree of commercialization. One of the most effective ways to improve the capacity of the batteries is to increase the working voltage [3,4]. For example, the working voltage of LiCoO_2_, one of the main cathode materials of LIBs for handheld terminals, has improved to 4.5 V up to now. However, it is necessary to ensure the high-temperature performance of the cathode, such as high-temperature storage and cycling characteristics, and the combined interval cycling performance under high applied voltage. The surface and internal structural stability of the cathode materials should be greatly improved, and more efforts should be made to match the used electrode and the electrolyte system.

The oxidation potential of the traditional organic carbonate solvent for LIBs is about 5.0 V (vs. Pt). However, the decomposition reaction begins at a voltage above 4.5 V due to the porous structure of LIBs, resulting in serious capacity attenuation of the battery at a high cut-off voltage [5,6,7]. During aging of the battery, the dissolved transition metal ions such as Ni^2+^, Co^2+^ and Mn^2+^ of cathode NCM or LiCoO_2_ would catalyze and accelerate the decomposition of electrolyte to form irreversible reaction products, resulting in an accelerated decrease in the capacity of LIBs [8]. For example, sulfone-based solvents have not yet been used in a large-scale prospective as a result of their poor compatibility with cathode materials due to their high viscosity, regardless of its relatively higher oxidation potential of above 5 V (vs. Li/Li^+^) [9,10]. Ethers-based electrolytes could offer lots of advantages like high ionic conductivity and coulomb efficiency [11,12], however, the disadvantages of ethers, such as the lower oxidation stability (electrochemical potential window of less than 4 V vs. Li/Li^+^), cannot be ignored, which prevents them from being used as ‘co-solvent’ of electrolytes [13,14]. Long-chain carbonates-based electrolytes and ionic liquid electrolytes can theoretically be used as high-voltage electrolytes due to their higher cycling stability [15], while the high viscosity would have significant negative effects on the ionic conductivity and the wettability of cathode/anode electrodes. In addition, other solvents having high melting points would cause significant deteriorations in low-temperature properties of LIBs. Therefore, the practical applications of the abovementioned solvents-based electrolyte systems have been severely hindered [16].

By adding a small amount of functional additive, such as film-forming additive, into the electrolyte system to form protective films at the cathode and anode interfaces, which could prevent the reaction between the cathode/anode and the solvent molecules, thus the structural damage of the positive and negative materials would be greatly reduced or even prevented. The rational approaches to selective functional additives have been proved to be more convenient than the development of new electrolytes [17,18,19,20].

**Figure 1:**
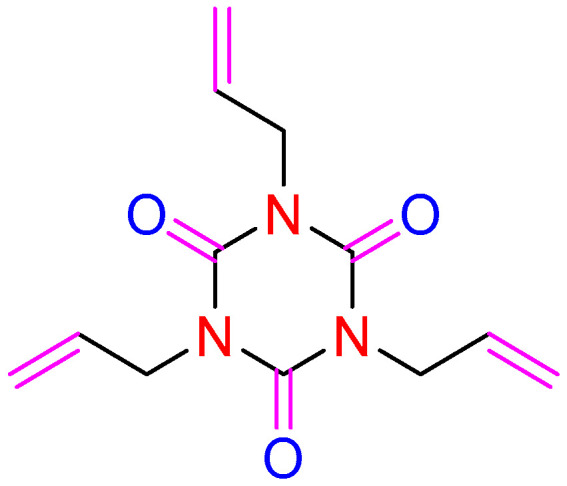
The chemical structure of TAIC.

To provide good protection against the deterioration at the cathodic interface, the formation of a protective polymer cathode/electrolyte interphase (CEI) film has been performed [21]. In recent years, the application of a large number of electrolyte additives including vinyl carbonate (VC), fluorides, new lithium salts, and other phosphorus (P)-/or boron (B)- or sulfur (S)-containing additives, and nitriles has been deeply investigated, and the possible effects and mechanisms have also been described. Interestingly, most of the additives exhibiting excellent cathodic protection ability toward high voltage LIBs possess double or triple bonds [22]. To the best of our knowledge, a crosslinking agent, 1,3,5-tri-2-propenyl-1,3,5-triazine2,4,6(1H,3H,5H)-trione (TAIC; Figure 1), has been widely used in the preparation of polymers [23,24], and the polymers fabricated by TAIC have shown relatively better thermostability.

In this paper, TAIC was selected and used as a multifunctional electrolyte additive to form a CEI at the cathodic interface, and the lithium cobalt oxide (LiCoO_2_)/electrolyte interface stability under high voltage was evaluated. The physicochemical properties of the battery were analyzed, and the underlying mechanisms were also investigated. The results indicated that TAIC-containing LIBs could maintain good performance under high voltage and high temperature conditions. This work may provide some useful references for the preparation of more stable LIBs due to the formation of high-quality CEI film in the battery.

## 2. Experimental Details

### 2.1. Preparation of the Electrodes

The additive 1,3,5-tri-2-propenyl-1,3,5-triazine2,4,6(1H,3H,5H)-trione (TAIC) was purchased from Alfa Aesar, Ward Hill, MA, USA. Main components of electrolyte such as lithium hexafluorophosphate (LiPF_6_), ethylene carbonate (EC), propylene carbonate (PC), diethyl carbonate (DEC), n-propyl propionate (PP), fluoroethylene carbonate (FEC) and 1,4-Dicyanobutane (ADN) were provided by ShanShan Advanced Materials (Quzhou) Co., Ltd., Quzhou, China. Anhydrous solvents were prepared by using 4A molecular sieve, then activated and mixed in a glove box filled with purified argon (Ar) in the absence of water and oxygen (below 1 ppm). The moisture and the content of free acid in the electrolytes of below 20 ppm and 50 ppm were measured by the Metrohm Coulomb Karl Moisture Tester and triethylamine titration, respectively. The codes of the electrolyte used are defined as Base-1# and TAIC-2#, respectively (Table 1).

The cathodic materials consisting of 96.0 wt.% LiCoO_2_ or NCM (which were provided by Tianjin B&M Science and Technology Co., Ltd.; Tianjin, China), 2.0 wt.% super P carbon black, and 2.0 wt.% poly(vinylidene fluoride) (PVDF) were prepared, respectively. LiCoO_2_ and conductive agents were homogeneously dispersed in N-methyl-2-pyrrolidone (NMP) to form a slurry with a viscosity of about 3000~6000 mPa·s. The slurry was coated onto an aluminum foil current collector, and then dried to obtain a cathode. The anodic material consisting of 96.3 wt.% graphite (which was provided by Jiangxi Zichen Technology Co., Ltd., Yichun, China), 1.0 wt.% super P carbon black, 1.5 wt.% styrene butadiene rubber (SBR), and 1.2 wt.% carboxy-methyl cellulose (CMC) was also fabricated. The active materials and conductive agents were homogeneously dispersed in deionized water to form a slurry with a viscosity of about 2000~4000 mPa·s, and an anode could be obtained by coating the slurry onto a copper foil current collector followed by air-drying.

A 2032-type coin cell was assembled by using NCM as the cathode, lithium metal as the anode, and a porous polyethylene (PE) separator of 9 μm thickness (W-Scope Co., Cheongju, Chungbuk, Republic of Korea) was placed between the cathode electrode and counter electrode. In order to evaluate the performances of the additive on the full battery, a wound 404,798-type battery was assembled by using LiCoO_2_ as the positive electrode, graphite as the negative electrode and a separator. Each coin cell and full battery used either reference Base-1# or functional additive electrolyte TAIC-2#.

### 2.2. Electrochemical Measurements

A three-electrode system with Pt metal as the working electrode, lithium metal as the counter electrode, and Pt as the reference electrode was assembled. Linear sweep voltammetry (LSV) of the three-electrode system was recorded over the voltage range from open-circuit voltage (OCV) to 7 V with a scanning rate of 1.0 mV/s with eight-channel Solartron potentiostat (model 1470E; Advanced Measurement Technology Inc.; Oak Ridge, TN, USA). AC impedances of coin cells and full batteries were collected over the frequency range 0.01~100,000 Hz with an amplitude of 5 mV. The coin cell of the NCM/Li system was charged–discharged for one cycle at a current rate of 0.1 C between 3.0 V and 4.4 V. The internal resistance of the battery was measured at 25 °C. High-temperature storage performance of the full batteries was measured using a temperature-controlled test chamber, which was kept at 85 °C. The long-term cycling performance of LiCoO_2_/graphite full batteries at 45 °C was evaluated in an incubator in the potential range 3.0–4.5 V at a current rate of 1 °C. Charge–discharge tests of LiCoO_2_/graphite full batteries were performed on a battery testing system (CT-4008W, Neware Battery Testing System, Shenzhen, China).

### 2.3. Characterization Methods

Field-emission scanning electron microscope (FE-SEM; Nova Nano SEM450; FEI Co., Hillsboro, OR, USA) was operated at an accelerating voltage of 15.0 kV to obtain surface morphologies of pristine electrode and tested electrodes. The harvested electrodes were rinsed three times with anhydrous dimethyl carbonate (DMC) to remove residual electrolyte, vacuum-dried in a chamber at 25 °C to remove excess DMC.

The structural changes in cathode can be investigated by X-ray diffraction (XRD; D8 ADVANCE). XRD patterns of the cathode were collected in the 2θ range of 15–89° at a scan rate of 0.01° S^−1^ using an X-ray diffractometer with Ni-filtered Cu Kα radiation generated at 40 mA and 40 kV. The chemical compositions of the cathode and anode surfaces of batteries were investigated by X-ray photoelectron spectroscopy (XPS; R3000, VG SCIENTA; VG Scienta, Uppsala, Sweden) with monochromatic Al Kα radiation under ultrahigh vacuum (≤2 × 10^−7^ mbar).

### 2.4. Computational Simulation

In recent years, the high-precision and multi-scale simulation of electrolyte additives for lithium-ion batteries could be implemented by various quantum chemistry theories such as density functional method and classical molecular dynamics (MD). Herein, the redox potential of the additive was obtained by calculating the highest-occupied-molecular-orbital (HOMO) and lowest-unoccupied-molecular-orbital (LUMO) energies by density functional method, and the additive potentiating electrolytes action in the battery can be justified theoretically. Molecular orbital energies of EC and DEC as electrolyte solvents, and TAIC as an additive, were calculated by Density Functional Theory (DFT) to propose the decomposition mechanism. The structural optimizations and frequency calculations of the solvent and additive molecules, and their solvated molecules with Li^+^ were carried out by DFT with the Triple-ζ Basis set 6-311++G (d, p).

## 3. Results and Discussions

### 3.1. Oxidation and Reduction Performance

As showing in Table 2, the HOMO level of TAIC was higher than those of electrolyte solvents. Furthermore, the LUMO value of TAIC additive (−0.56 eV) was somewhere in between EC (−0.6) and DEC (−0.42). TAIC was confirmed as a suitable additive for cathodes toward high power applications. The preferential oxidation of TAIC additive can be expected, and a stable interfacial layer on the cathode surface would be formed.

**Figure 2:**
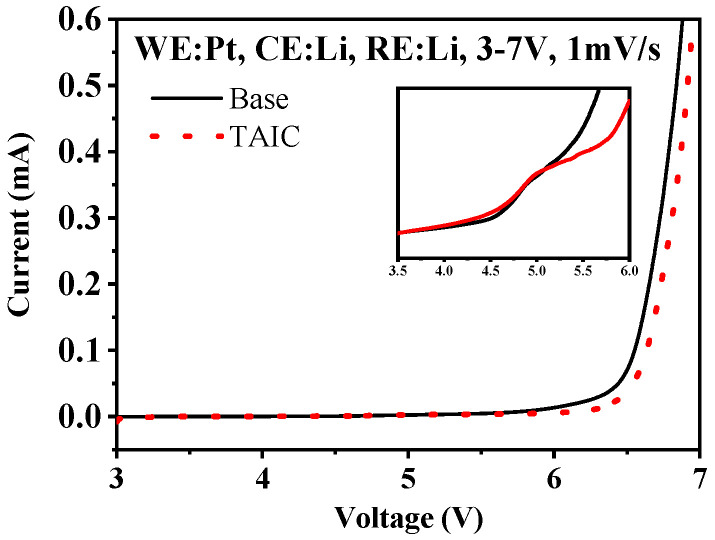
LSV curves of the three-electrode setup in different electrolytes (Inset: Base-1# and TAIC-2#) (The scanning voltage ranged from OCV to 7 V, and the scan rate was 1 mV/s).

To verify the theorical hypothesis, LSV was performed to investigate the oxidation potential in different electrolytes with and without TAIC. As shown in Figure 2, an apparently increased oxidation current can be observed at the potential of ~4.3 V in the electrolyte containing TAIC, confirming that TAIC was oxidized preferentially. Oxidation current slowly increased when the voltage was swept to above 5.0 V in the TAIC-containing electrolyte, while the oxidative current sharply increased in the electrolyte without TAIC, indicating that the electrolyte without TAIC suffered severe decomposition. Obviously, the preferential oxidation of TAIC would efficiently suppress the decomposition of conventional electrolytes.

### 3.2. Influence of Additive TAIC on Electrochemical Performance

**Figure 3:**
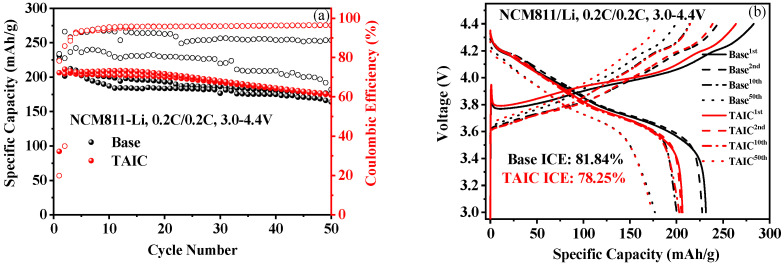

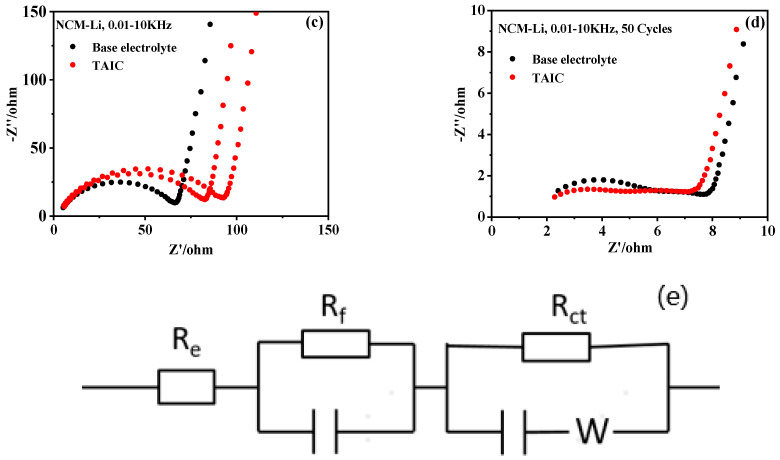
(**a**) The cycle curve of NCM-Li half-cell (The solid balls represent the cyclic capacity attenuation, and the hollow balls represent the charging-discharging efficiency in the cyclic process); (**b**) The charge–discharge curve during the charging process; (**c**) Representative EIS Nyquist plots of NCM-Li half-cells prepared with different electrolyte models measured after the first discharge–charge cycle at 0.1 C; (**d**) Representative EIS Nyquist plots of NCM-Li half-cell with different electrolyte models after the 50th discharge–charge cycle; (**e**) An equivalent circuit model.

Cycle curves of a NCM-Li half cell (a) and charge–discharge curves were recorded (Figure 3). The AC impedance (Figure 3c,d) and the equivalent current model (Figure 3e) of NCM/Li systems are also displayed. As shown in Figure 3b, the difference of the first charging–discharging capacity of the cell in TAIC-containing electrolyte was greater than that in a basic electrolyte; the first efficiency (ICE) of TAIC-containing electrolyte was 78.25%, lower than a base electrolyte (ICE: 81.84%), indicating that a solid electrolyte protective film might be formed by consuming Li^+^ during the charging process. After five cycles, the charge–discharge efficiencies stably maintained at ≥99%, which was much higher than those in the basic electrolyte (Figure 3a). Furthermore, the relatively better overall cycling performance of the NCM/Li half-cell with a TAIC-containing electrolyte could be observed. The impedance of the NCM/Li half-cell increased if the TAIC was added to the electrolyte. With an increase in the charge–discharge cycle, sufficient electrolyte infiltration occurred. Active edge sites of layered ternary material (NCM) gradually increased, which correspondingly promoted the charge transfer efficiency, and the overall impedance tended to decrease in comparison with the initial value. Due to the film-forming effect of additive TAIC, the increase rate in interfacial impedance was relatively smaller than that with base electrolyte, again indicating that the addition of TAIC to the electrolyte was beneficial.

**Figure 4:**
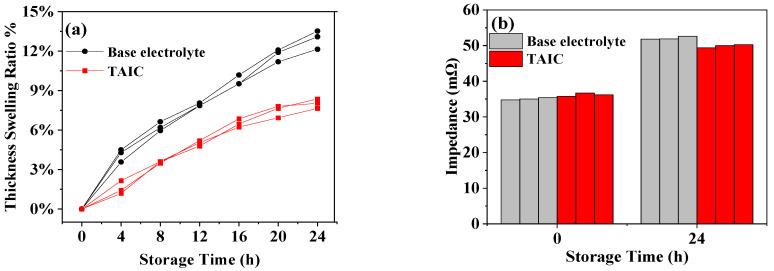
The full-cell storage performance of 85 at °C: (**a**) Thickness swelling; (**b**) Variation in impedance.

Figure 4 shows the storage performance of full-cell at a high temperature of 85 °C. After adding TAIC, the initial impedance of the full-cell was around 35.0 mΩ, which was higher than with a basic electrolyte. With the increase in storage period (24 h), the thickness expansions (~6%) of the cell with TAIC was relatively smaller than those (~13.5%) with basic electrolyte. In particular, the impedances of a full-cells with TAIC-containing electrolytes were about 50.0 mΩ, which were smaller than those (~52.0 mΩ) with base electrolytes, indicating that the addition of TAIC could alleviate the oxidation decomposition of the electrolytes by the formation of surface passivation film at high temperatures and high working voltage.

**Figure 5:**
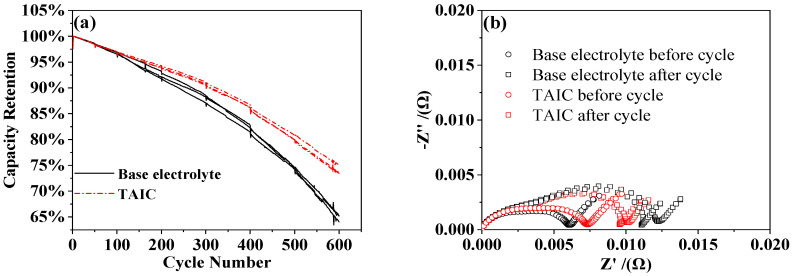
Cycle performance of full-cell at charge/discharge rate of 1 °C at 45 °C: (**a**) Capacity retention rate; (**b**) EIS Nyquist plots of LiCoO_2_ vs. Li^+^/Li EIS with a different electrolyte before and after the cycle.

Figure 5 shows the cycle performance of polymer full-cell at 45 °C. After 600 cycles, the capacity retention rate of the full-cell without TAIC additive was about 65.3%, while the capacity retention rate of the full cell with 0.5 wt.% TAIC additive in the electrolyte increased to 75.8%. The positive interface impedance of the three-electrode setup was measured by EIS, and the initial positive interface impedance after adding TAIC was higher than that with base electrolyte, confirming that the TAIC additive was beneficial to the formation of a protective layer at the LiCoO_2_ interface, which would increase the interface impedance. After 600 cycles, the positive interface impedance of the full-cell with a TAIC additive was smaller than that with a base electrolyte, indicating that TAIC-assisted film formation played a role in protecting the cathode interface and reducing the capacity fading. Further oxidization and decomposition of the electrolyte by the cathode was efficiently prohibited by TAIC additive, which would have a significant impact on the cycle performance of the battery.

**Figure 6:**
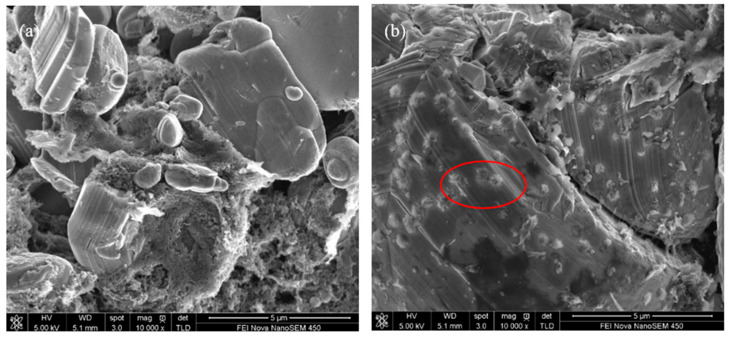

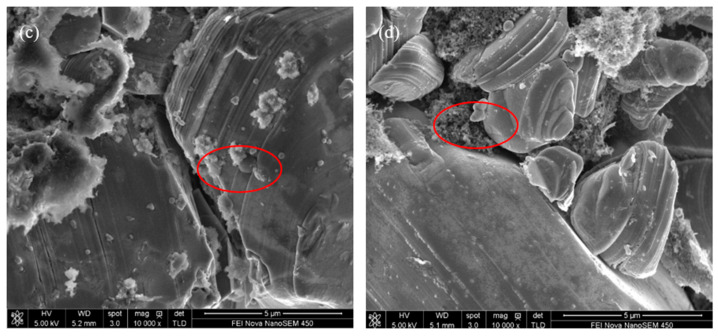
SEM images of LiCoO_2_ cycled with a base electrolyte: (**a**) After the first cycle and; (**b**) after 600 cycles; SEM images of LiCoO_2_ cycled with the electrolyte containing 0.5% TAIC; (**c**) after the first cycle; (**d**) after 600 cycles. The areas marked in red indicate the byproducts observed.

SEM characterizations of the electrodes after cycling were performed, and the effectiveness of TAIC for improving the cycling performance of the batteries was confirmed (Figure 6). After the first cycle, the surface morphological changes of LiCoO_2_ after cycling were monitored. More byproducts are displayed on LiCoO_2_ particles with the TAIC-containing electrolyte, while the smoother surface of LiCoO_2_ particles cycled in the base electrolyte could be observed. However, higher side reaction product contents on the interface crack are exhibited along with the more cracks at the positive interface with the base electrolyte after 600 cycles. The SEM results were consistent with the cycle performances of the batteries previously discussed.

**Figure 7:**
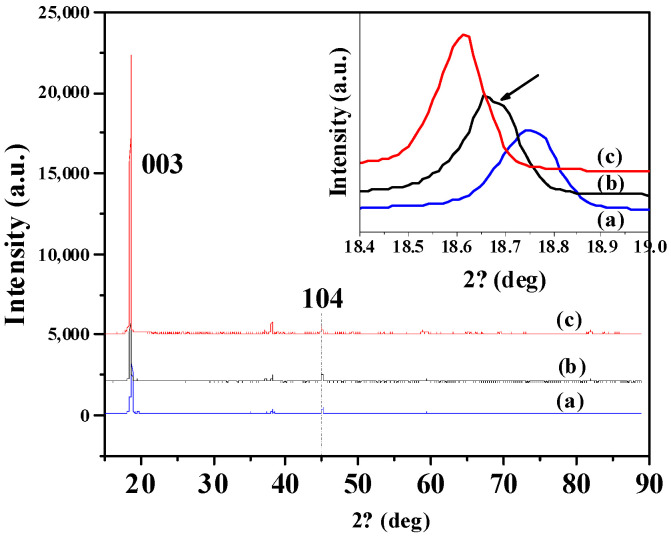
XRD patterns of LiCoO_2_ powders: (**a**) Pristine LiCoO_2_; (**b**) cycled in a base electrolyte at 45 °C; (**c**) cycled in the electrolyte containing 0.5 wt.% TAIC at 45 °C.

Figure 7 shows the structural changes of pristine LiCoO_2_ powder, and the LiCoO_2_ powders from the cathode electrode of full-cell with a base electrolyte and with a 0.5 wt.% TAIC-containing electrolyte after cycling at 45 °C. The strong peak of (003) at 18.75° for pristine LiCoO_2_ powder could be observed, and the peaks at 18.65° and 18.60° of LiCoO_2_ powders from the cathode electrode of full-cell with a base electrolyte and with a 0.5 wt.% TAIC-containing electrolyte could be detected. Obviously, the sharp splits in 003 peak of the electrode powder cycled without a TAIC additive confirmed its higher transverse effect, while the electrode powder with the electrolyte containing 0.5 wt.% TAIC showed no significant changes in the peak intensity in comparison with that pristine LiCoO_2_ powder, indicating that the crystal structure of the LiCoO_2_ powder was easily subjected to damage under high temperatures and high voltage, while the addition of TAIC to the electrolyte could protect the structure and function of LiCoO_2_ by the formation of surface passivation films.

**Figure 8:**
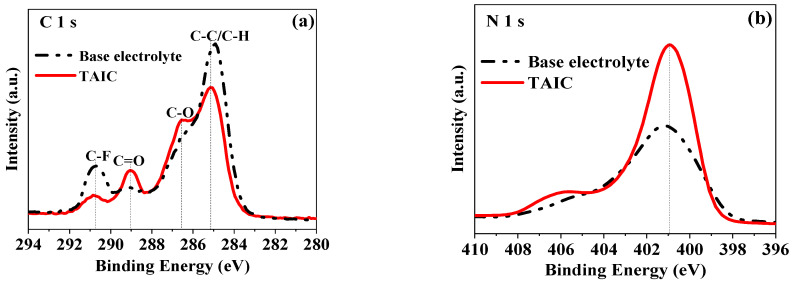
XPS spectra of LiCoO_2_ after cycling in base and 0.5 wt.% TAIC-containing electrolytes: (**a**) C1s; (**b**) N1s.

Figure 8 presents the XPS patterns of LiCoO_2_ electrodes after cycling at 45 °C in base or 0.5 wt.% TAIC-containing electrolytes. In C1s spectra, relatively weaker peaks of C-C (285.1 eV) and C-F (290.8 eV) of the LiCoO_2_ electrode in TAIC-containing electrolyte could be observed, while relatively stronger peaks of C=O (289.0 eV) and C-O (286.6 eV) of the electrolytes electrode in TAIC-containing electrolyte could be detected [2]. The results suggested that more decomposition products such as alkanes and F-containing compounds would be produced for LiCoO_2_ in a base electrolyte, and alkoxy-containing molecules would be formed in TAIC-containing electrolyte. In N1s spectra, the peak of C-N (400.9 eV) corresponding to the LiCoO_2_ electrode in both electrolytes could be observed, which might be attributed to C-N bond-containing molecules. The stronger peak at 400.9 eV in TAIC-containing electrolyte can be possibly ascribed to the introduced TAIC. Therefore, more lithium alkyl carbonates and polycarbonates were deposited on LiCoO_2_ in a base electrolyte, while the protective SEI film formed on LiCoO_2_ particles by TAIC during the initial charge process was effective in prohibiting the possible interactions between LiCoO_2_ and the electrolyte during the following cycling processes.

## 4. Conclusions

TAIC is easily oxidized, potentially at about 4.25 V. However, a film on the cathode (LiCoO_2_ and NCM) will be formed in the present of TAIC in the electrolyte, which can effectively protect the positive interface due to the larger film-forming impedance. The LiCoO_2_/Gr polymer full-cell further confirmed the film-forming reaction of cathode in TAIC, and the alkoxy-containing byproducts formed can stably prohibit further reaction between the LiCoO_2_ powder and the electrolyte, although the initial impedance was relatively larger. By adding 0.5 wt.% TAIC to the electrolyte, the battery showed better high-temperature storage performance and cycle performance, and thickness swelling decreased to about 6% after 24 h storage at 85 °C. In addition, the capacity retention of the battery at 45 °C increased 10% by adding TAIC when it was cycled under 4.5 V after 600 cycles. The improvement could be attributed to the formation of a stable and protective SEI film on LiCoO_2_ due to the preferential oxidation of TAIC. The SEI film containing the decomposition products of TAIC on the LiCoO_2_ was thicker. The further decomposition of electrolyte was efficiently suppressed, and the destruction of LiCoO_2_ was therefore greatly prevented. Furthermore, the addition of TAIC to the electrolyte could decrease the increase in interface impedance after cycling, especially with regard to the impedance of LiCoO_2_ cathode.

## Figures and Tables

**Table 1:** The compositions and codes of various electrolytes.

Code	Solution				Additive/wt.%			Lithium Salt/mol L^−1^
	EC	PC	DEC	PP	FEC	ADN	TAIC	LiPF_6_
Base-1#	20	20	20	40	3	3	0	1.2
TAIC-2#	20	20	20	40	3	3	0.5	1.2

**Table 2:** HOMO and LUMO levels of TAIC, EC and DEC.

Organic Molecules	TAIC	EC	DEC
HOMO (eV)	−7.86	−8.47	−8.02
LUMO (eV)	−0.56	−0.6	−0.42

## Data Availability

Not applicable.

## Sample Availability

Samples of the compounds are not available from the authors.
